# Resilience of Maize to Environmental Stress: Insights into Drought and Heat Tolerance

**DOI:** 10.3390/ijms26115274

**Published:** 2025-05-30

**Authors:** Huaijun Tang, Lei Zhang, Xiaoqing Xie, Yejian Wang, Tianyu Wang, Cheng Liu

**Affiliations:** 1 Institute of Crops Research, Xinjiang Academy of Agricultural Sciences, Urumqi 830091, China; tanghuaijun83@sina.com (H.T.); 18999224030@163.com (L.Z.); 13565990757@163.com (X.X.); wangyejian0815@163.com (Y.W.); 2 Institute of Crop Sciences, Chinese Academy of Agricultural Sciences, Beijing 100081, China

**Keywords:** abiotic stress, drought resistance, heat stress, climate adaptation, physiological responses

## Abstract

Maize (*Zea mays* L.) is a staple cereal crop worldwide, but its productivity is significantly affected by extreme weather conditions such as drought and heat stress. Plant growth, physiological processes, and yield potential are all affected by these conditions; as such, resilient maize crops are required to tackle these abiotic challenges. With an emphasis on morphological, physiological, and biochemical reactions, this review paper investigates the processes that underlie resistance to certain environmental challenges. Features including deep root systems, osmotic adaptations, and antioxidant enzyme activity help maize withstand drought. Activation of drought- and heat-responsive genes, accumulation of osmoregulatory compounds, and changes in membrane fluidity are all components of abiotic stress tolerance. Likewise, improved transpiration efficiency, modified photosynthetic processes, and improved heat shock proteins are used to produce heat resistance. Enhancing resilience requires progress in breeding methods, genetic engineering, and agronomic techniques, such as the use of stress-tolerant cultivars, biotechnology interventions, and climate-smart agriculture tactics. A special focus was given to cutting edge technologies like CRISPER-Cas9-mediated recent advances in heat and drought resistance. This review sheds light on recent studies and potential avenues for enhancing resilience to harsh climatic conditions, guaranteeing food security in the face of climate change.

## 1. Introduction

Air temperatures have increased since the turn of the 20th century and are predicted to continue to climb due to climate unpredictability. High-temperature stress (HTS), which causes major harm to plants, may be caused by these extreme conditions [1,2]. Extreme drought and heat stress severely reduce crop productivity by disrupting key physiological processes like photosynthesis, pollination, and grain filling. These stresses are major threats to global food security, especially for staple crops like maize, wheat, and rice. As climate change intensifies, the frequency and severity of such events are expected to rise, putting additional pressure on agricultural systems. Therefore, given the current agroclimatic conditions, food security has emerged as a critical challenge [3,4,5]. High day and night temperatures are endangering the world’s agricultural production system, according to climate models [6]. Globally, this leads to a decrease in maize crop yield and productivity [7]. Maize is one of the most widely cultivated crops globally, serving diverse purposes as a staple food source. Efforts to improve its yield, quality, and stability across various growing conditions remain a primary focus [5,8]. It is the most important cereal crop in the world and is used as a raw ingredient in many culinary and feed businesses.

Heat stress is one of the growth-limiting variables that significantly affect maize’s growth and nutritional content at various stages of development [9,10]. The development of better breeding practices is crucial for raising maize yield and quality, since many abiotic challenges, such as heat stress and drought stress, occur simultaneously [11]. Further research is vital for creating maize genotypes resistant to drought stress and high temperatures. Several experts consider stomatal conductance a key indirect parameter for selecting heat-tolerant crops [12]. Similarly, osmoprotectants and chaperone proteins play a vital role in maize’s adaptive response to heat stress and other environmental stressors. Additionally, proteins associated with leaf senescence enhance maize’s resilience to both heat and drought stress [13]. It is possible to create maize hybrids that are resistant to heat and drought stress by introducing these features into locally adapted hybrids through possible donor hybrids. Moreover, identification of donor genotypes possessing favorable traits is important in heat stress breeding programs [14]. Cereal plants of the grass family provide nearly half of humanity’s total caloric intake and offer more nutrition than any other food group. Although more than a dozen cereal crops are consumed as food, for example, wheat, maize, and rice dominate human diets, accounting for 94% of total cereal consumption. Maize is the third most important cereal crop contributing to global food security [15]. Cereal consumption varies significantly across regions, with rice being the predominant crop in Central Asia, the Middle East, North and South America, and Europe. While maize, commonly known as corn, is preferred in Southern and Eastern Africa, Central America, and Mexico, rice is the predominant cereal in Asia.

Maize is a good source of fiber, several B vitamins, and essential minerals. However, it is generally low in calcium, folate, and iron, and lacks certain nutrients, including vitamin B12 and vitamin C. Certain foods or components in the diet, such as vegetables, tea (like oxalates), coffee (like polyphenols), eggs, and milk (like calcium), might limit the absorption of iron, especially the nonheme iron found in maize [16]. In countries where anemia and iron deficiency are considered moderate or severe public health problems, the fortification of maize flour and cornmeal with iron and other vitamins and minerals has been used to improve micronutrient intake and prevent iron deficiency (WHO, 2009). As maize is a vital staple food crop and contributes significantly to global food security, efforts were made to fortify maize. The development of Zn- and Fe-enriched cultivars are great examples of fortification through biotechnological approaches and conventional breeding [17,18].

The aim of this review is to provide a comprehensive understanding of the mechanisms underlying maize resistance to drought and heat stress, focusing on morphological, physiological, and biochemical responses. The objective is to highlight recent advances in breeding strategies, genetic engineering, and agronomic practices that enhance abiotic stress tolerance, with the ultimate goal of supporting the development of climate-resilient maize varieties to ensure food security under changing climatic conditions.

## 2. Major Challenges Posed by Abiotic Stress in Maize

The increasing ecological impacts of climate change and the rising competition for environmental resources due to population growth underscore the challenges abiotic stress poses to plant growth and development. Heat and drought stress can significantly reduce maize yields by affecting plant growth, pollination, and kernel development. These stresses often occur together and worsen each other’s impact. During critical growth stages like flowering and grain filling, even short periods of high temperature or water shortage can cause substantial yield loss. Maize is particularly sensitive to water deficits during silking, leading to poor kernel set. Overall, the combination of heat and drought can lead to high yield losses, depending on the severity and timing of the stress [19,20,21]. Agricultural production is expected to be most affected by climate change, particularly in low-latitude regions where developing nations are concentrated [22]. The negative consequences of rising carbon dioxide and high temperatures will compel researchers to develop effective adaptation strategies [23]. These limitations on the world’s food supply and environmental balance promote the study and creation of climate-smart and climate-resilient crops [24].

All research on environmental stressors or abiotic elements that can cause stress to a range of species is included in the field of plant abiotic stress [25]. Extreme and low levels of light, UV-B and UV-A radiation, temperature extremes (cold and freezing), water extremes (drought, flooding, and submersion), chemical factors (heavy metals and pH), salinity from too much Na^+^, a lack or surplus of vital nutrients, gaseous pollutants (ozone and sulfur dioxide), mechanical factors, and other less common conditions are some examples of these stressors. A range of physiological interactions is expected, as multiple stresses such as heat and drought often occur simultaneously in field conditions, leading to unique effects that cannot be predicted from individual stressors alone, thereby requiring innovative, tailored solutions [26].

Plants, being deeply rooted in their environment, must continuously adapt to changing conditions influenced by various environmental factors. When these factors exceed optimal levels, they can cause abiotic stress, challenging the plant’s growth and survival. Determining how plants sense various stressors, how early signals are transduced inside the plant, what variety of response pathways they trigger, and how they are genetically defined is a major challenge in abiotic stress biology [27]. Depending on a plant’s genetic makeup and adaptive response, environmental stimuli, whether biotic or abiotic, might present a challenge or stress. An environment suitable for one genotype of plant may not be for another. Numerous impacts in response to the environment are provided by particular genotype × environment interaction combinations [28,29].

## 3. Significance of Understanding and Improving Maize Adaptability to Drought and Heat

Climate factors such as temperature, precipitation, and humidity influence crop development, with the impact of climate change on yields varying by crop type and region. Crops can experience growth suppression due to abiotic stressors including drought and severe temperatures [30]. These stressors’ main characteristics include the potential to lower crop vigor, impede growth and development, and lower crop output. Extreme weather events, including heat waves and droughts, severely affect maize productivity. These elements hinder maize growth, damage crops during drought, and drastically lower output [31]. As temperatures rise and rainfall deficits grow, the frequency of droughts is expected to increase. Crop yields are most severely impacted by drought stress during the reproductive or growth stage. Plant growth, reproduction, and physiology are all negatively impacted by drought, which has a significant impact on agricultural output [32]. Field experiment data from 1980 to 2015 indicate that drought, causing approximately 40% water loss, reduced maize yields by 39.3% and wheat yields by 20.6% [33]. In both dry and non-arid areas, maize was more vulnerable to drought than wheat, particularly during the reproductive stage. Drought stress can significantly reduce kernel number, ear size, and overall plant growth. During the vegetative stage, up to V12, drought stress often affects the final maize yield. Maize is particularly vulnerable to stress during fertilization and pollination, with yields potentially decreasing by 3–4% per day if drought stress persists for two weeks before pollination, causing the plant to wilt. Yield losses might reach 8% per day, depending on the degree of stress experienced during the formation of silk and pollen. Furthermore, yield losses might amount to as much as 6% per day if drought stress continues for two weeks following silking.

**Figure 1:**
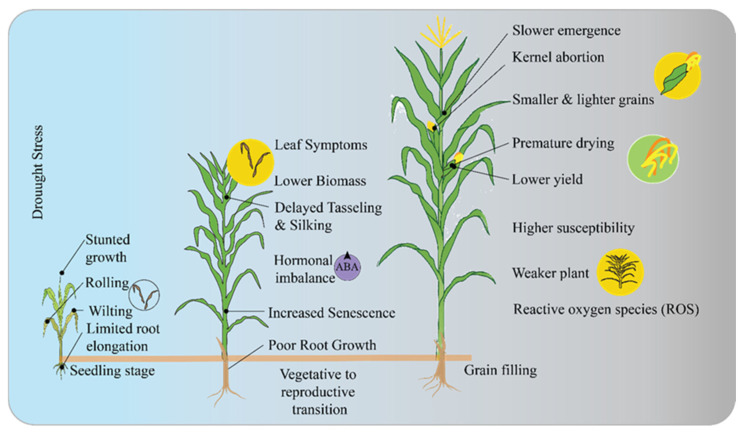
Maize response to drought stress at critical growth stages. The symptoms such as stunted growth, wilting, hormonal imbalance (ABA), and reduced yield. It illustrates how drought impacts plant development from seedling emergence to grain filling, leading to issues like slower emergence and kernel abortion.

In maize, pollination refers to the transfer of pollen from tassels to ears. When pollen germinates on the silk, it forms a pollen tube that delivers genetic material to each ovule, leading to kernel formation. However, extreme heat stress and drought can disrupt the synchronization of silk emergence and pollen availability, affecting fertilization (Figure 1). Additionally, it can dry out exposed silk, which prevents it from absorbing pollen grains. In the meantime, drought stress is typically accompanied with high-temperature damage during the pollination phase in maize, though it can also happen on its own. Temperatures exceeding 35 °C, combined with low relative humidity, generally dry exposed silk but have little effect on its elongation. Conversely, low relative humidity and temperatures above 32 °C increase the risk of pollen damage or loss. Studies suggest that prolonged exposure to temperatures above 32 °C can negatively affect moisture levels and grain filling [34]. Fortunately, pollen release typically occurs between early and mid-morning, ensuring a daily supply of fresh pollen until maturation and production are complete.

Every plant has a range of ideal temperatures for growth, and temperatures outside of this range hinder the development and growth of the plant. One of the most dangerous abiotic stresses is the slow rise in the yearly mean temperature and heat. Ultimately, rising temperatures can significantly impact crop growth and development, which could influence agricultural products’ output and quality [35]. In cooler climates, some crops may benefit from rising temperatures; however, overall, higher temperatures reduce yields and negatively impact crop productivity. The first application of the Crop-Environment Resource Synthesis (CERES) maize model was to estimate historical variations in maize yield over around 200 sub-Saharan African regions [36,37]. The study modeled a fictitious future in which maize yields in sub-Saharan Africa would decline more with a 2 °C rise in temperature than with a 20% drop in precipitation. Compared to other organs, reproductive organs have a much lower temperature threshold for heat stress injury [38]. The synthesis of viable pollen, its transfer to the male gamete’s germ cell, and the start and maintenance of embryo and endosperm development are all necessary for maize to mature into a kernel. High temperatures are linked to lower production because they reduce grain weight and number, especially during reproductive growth. Fewer ovules will mature into kernels when fertilized at high temperatures. Furthermore, heat stress can alter the shape and physiological function of the tassels, the reproductive structures responsible for pollen production [10]. Rising temperatures and shifting precipitation patterns, driven by increased greenhouse gas emissions, pose a significant threat to crop yields and global food security. As temperatures rise, drought intensifies due to rapid moisture loss from soil surfaces and plant tissues, while high temperatures can also directly damage crops [39]. Food security depends on agriculture, which is severely impacted by heat stress and drought, either separately or in combination. Prolonged drought and extreme climate variability significantly reduce crop yields and increase losses during critical stages of maize growth and development. Maize yields have been steadily declining due to irregular rainfall patterns, while drought conditions have also led to a reduction in maize cultivation areas. Additionally, the combined effects of heat and drought during critical growth stages can significantly impact crop productivity. These abiotic stressors are widely recognized for influencing the growth and spread of weeds, insects, and diseases, often leading to the emergence of harmful pests [40]. Through its effects on photosynthesis [41], drought reduces crop growth and yield by inducing wilting, slowed growth, delayed leaf emergence, and reduced leaf area, particularly during the seedling stage [42]. In maize, yield is decided during the flowering stage due to the close association between pollen release and the growth of maize silk; further, there is a close relationship between the final yield and the ASI (Anthesis–Silking Interval). Consequently, drought stress during blooming inhibits the growth of maize silk and lowers production [43,44].

## 4. Drought Resistance in Maize

### 4.1. Root Architecture and Water Uptake Efficiency

**Figure 2:**
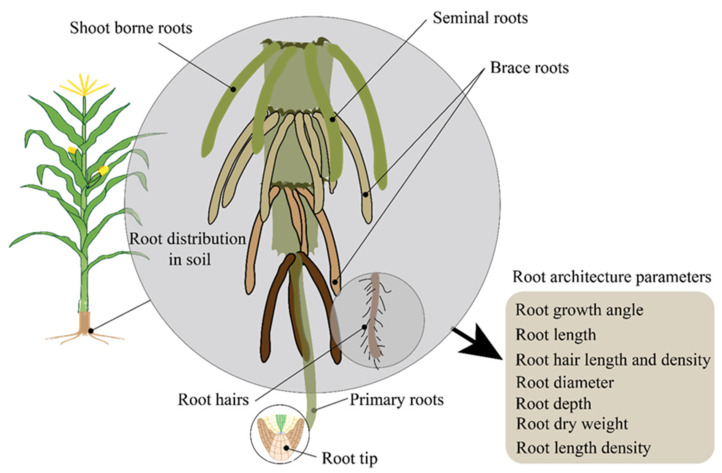
Root architecture parameters regulating drought tolerance in maize. The maize root system and different types of rooting in maize.

A promising strategy for enhancing crop drought tolerance is breeding crops with root systems optimized for efficient water uptake [45]. Given the complex relationship between root traits and diverse hydrological conditions, modeling provides essential insights for trait-based selection. In one study, the effect of root architecture was examined [46]. Other studies have investigated whether a universally drought-adapted root system ideotype exists or if water uptake efficiency depends on specific hydrological conditions by integrating a root architecture model with a soil–hydrological model [47,48]. To achieve this, the transpiration of 48 root designs was modeled across sixteen drought scenarios, varying in soil texture, rainfall patterns, and initial soil moisture availability. The findings indicate that hydrological conditions directly influence the efficiency of water absorption by root structures. It is not always the case that deep and dense root systems are better at absorbing water. In a different study, findings showed that identifying root systems with optimal functionality requires more than just architectural description [49]. When rainfall is the primary source of water during root system development, root density, especially near the soil surface, becomes crucial for optimizing soil moisture uptake. Therefore, we conclude that trait-based root breeding should prioritize root systems adapted to the hydrological conditions of the target environment. For instance, some root-related parameters are presented in Figure 2. Root architecture plays a vital role in a plant’s resource-foraging strategy and is a key determinant of productivity [50,51]. The key factors influencing overall water uptake are shown in Figure 2.

The efficiency (units of nutrient acquired per unit of resource invested by the plant) with which various root architecture forms obtain nutrients from soil have been attempted to be quantified using theoretical analysis. According to these findings, highly branched (dichotomous-like) root structures could efficiently capture immobile ions (like phosphate) from limited soil volumes. This capability paves the way for the targeted exploration of local soil volumes with the highest level of precision [52,53]. Additionally, these results indicate that herringbone-like root systems with coarser, sparser branches are better at capturing mobile nutrients over broad soil volumes (maximizing scale). The latter are better at examining spatially varied soil, but they can be costly in terms of carbon and may only be preferred when development is constrained by the soil’s resource availability [53]. These analyses offer a valuable theoretical framework for studying root architecture. However, root systems were depicted as ineffective structures that were uniformly nutrient-supplied, and the conclusions were solely based on diffusion theory. Plant root systems are typically not insensitive to their surroundings, and soils are not all the same. This raises the question of whether such generalizations hold true for real-world root systems, which interact dynamically with soils that vary across space and time. Locally elevated absorption kinetics and locally accelerated root proliferation are two well-known plasticity mechanisms that allow root systems to adapt to their varied environment [48]. Individually, these two processes can play a crucial role in nutrient acquisition, helping to mitigate supply inconsistencies [54].

### 4.2. Osmotic Adjustment and Accumulation of Compatible Solutes

**Figure 3:**
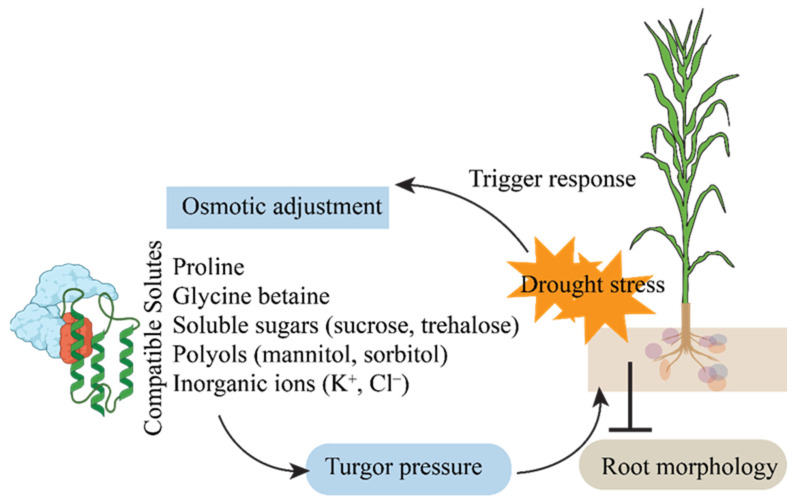
Illustration of the adaptive mechanisms of maize under drought stress, including osmotic adjustment, maintenance of turgor pressure, and modifications in root morphology.

In response to drought, plants either sustain water absorption or reduce water loss (stomatal conductance). Osmotic adjustment (OA), a biochemical process that aids plants in adapting to dry and salty environments, facilitates the latter process within plant cells [55]. The quantity of osmotically active compounds in the cell increases because of OA [56]. In leaf tissue and other metabolically active cells, this increase in solutes results in a greater negative osmotic potential, which can enhance the level of cell hydration and preserve turgor. In other words, if OA happens, plants can continue their metabolic activities and live longer in drying soil. OA has improved productivity and growth of several crop cultivars under drought stress (Figure 3). OA can be caused by a variety of chemicals, such as organic acids, amino acids, carbohydrates, and inorganic cations and anions (Figure 3).

The accumulation of certain solutes with protective properties is frequently linked to OA. These hydroxyls (−OH), a group rich in compatible solutes, which include sugars, cyclitols, proline, and glycine betaine, can build up in the cytoplasm and aid in preventing dehydration of cellular membranes, proteins, and enzymes [57]. However, it is crucial to understand that in many species, OA is primarily caused by the accumulation of several solutes, and that individual solutes do not significantly contribute to OA. OA is typically a gradual process that is sensitive to the timing and severity of stress since it necessitates metabolism or absorption of solutes.

There is some indication that studies have underestimated leaf relative water content (RWC) when assessing OA, which is on top of the intrinsic heterogeneity in OA expression. An accurate evaluation of the relative capacity for OA in various plants depends on the proper monitoring of plant water status, such as RWC. When threatened by desiccation due to drought or external lowering of the osmotic pressure, such as increase in soil salinity, most organisms increase the cellular concentration of osmotically active compounds, known as compatible solutes [58,59,60]. At high quantities, the accumulating chemicals are “compatible” with regular cellular metabolism [61]. The idea that suitable solutes could take the role of water at the surface of proteins, protein complexes, or membranes stems from the fact that they are often hydrophilic [62]. The term “compatible solute” carries a physiological connotation but does not explicitly define the functions these solutes perform. Although the biochemical processes by which suitable solutes provide protection are currently unclear, this does not necessarily mean that efforts to create transgenic plants that promote metabolite accumulation are off the table.

Many studies supported the concept of osmotic adjustment in maize to overcome the drought stress conditions. Two tropical lowland maize populations were used in a study to understand components of its genetic variance and heritability in maize [63]. The findings showed that more genetic variation was detected with data collected at the flowering stage, when water stress was more severe, than at the vegetative stage [63]. Later in a different experiment, osmotic adjustment was examined in a group of maize hybrids at different stages of growth. The results showed a positive tendency was observed between osmotic adjustment and phenotypic stability [64]. OA is used as a critical parameter in maize to measure the effect of drought on plants [65,66]. OA of cells helps to conserve the water balance of the plant, and this adjustment is generally achieved through increased amounts of various common solutes. Such evidence shows that maize crops have showed tolerance to water deficit through the mechanism of osmotic adjustment [67], and therefore genes related to OA should be mined and breeding goals can be adjusted accordingly.

### 4.3. Role of ABA (Abscisic Acid) in Stomatal Regulation

Abscisic acid (ABA), a plant hormone, is essential to plant viability. ABA is involved in developmental processes and rapidly accumulated in plants is observed in response to abiotic stress [68]. When plants encounter abiotic stress, they rapidly trigger the ABA signaling pathway, leading to the activation of transcription factors that respond to ABA and the subsequent expression of ABA-responsive genes [69]. Protein kinases and phosphatases play pivotal roles in ABA transduction in plants. During stress conditions, ABA accumulation starts and these molecules bind to resistance/regulatory components of ABA receptor (RCARs). Mutant plants that lack components of ABA signal transduction or ABA production exhibit extreme drought susceptibility. Our knowledge of ABA signal transduction has improved over the past ten years since the ABA receptor genes were discovered [70,71]. Numerous investigations using genetic, physiological, biochemical, chemical biology, and evolutionary methodologies were used to advance research about role of phytohormones in stomatal regulation. It has been well documented that abscisic acid-induced stomatal closure during abiotic stress [72]. ABA regulates stomatal closure by a chemical signal that can activate the metabolic process through a series of signaling cascades [73]. Furthermore, recent research has demonstrated that ABA signaling, also known as basal ABA signal transduction, plays a crucial role in regulating stomatal control, plant development, and metabolic pathways in stress condition [27,74]. Ion efflux from guard cells mediates stomatal closure triggered by abscisic acid. Stomatal closure is the outcome of ion efflux, which also causes osmotic water efflux and a decrease in the guard cells’ volume and turgor. S-type and R-type anion channel activation are key factors in stomatal closure [75,76]. Anion efflux from guard cells is mediated by these channels, and they also depolarize the plasma membrane. These channels triggers depolarization-activated potassium (K) efflux channels. Such channels have an important role in regulation of anion efflux from guard cells and depolarize the plasma membrane. Finally it activates potassium efflux channels responsive to depolarization [76]. The activity of both potassium (K) efflux channels and S-type anion channels facilitates efficient solute release from guard cells and enables stomatal closure. ABA is a plant hormone that can regulate the physiology metabolism under drought conditions in maize. For instance, exogenous ABA was found effective remedies for maize ear dysplasia at grain filling stage under drought stress [77]. Maize Calmodulin-like 3 Gene (*ZmCML3*) positively regulates drought resistance in maize. The gene was targeted after being induced by ABA [78]. Therefore, the ABA pathway is extensively targeted in different crops to understand the drought mechanism. The genes and pathways related to ABA synthesis and signaling are hot topics for research related to the effects of drought on maize.

### 4.4. Molecular and Genetic Approaches for Drought Tolerance

Understanding the molecular mechanisms of drought-resistant genes in maize is crucial. This knowledge helps develop resilient varieties. Such varieties can address challenges caused by drought. This entails identifying potential genes linked to drought resistance, as well as examining how these genes express themselves during drought stress. Numerous techniques, like genome-wide association studies (GWAS), QTL mapping, comparative genomics, and gene expression profiling, have made it easier to identify drought-resistant genes in maize. Several key drought-resistant genes play a crucial role in drought tolerance by regulating osmotic balance, enhancing water-use efficiency, and activating stress-induced signaling pathways [79]. Determining the function of drought-resistant genes in stress tolerance requires an understanding of how these genes are expressed during drought. The regulation of these genes during dehydration and their role in preserving cellular homeostasis are clarified by gene expression investigations. Table 1 shows some recently identified genes and their role in drought resistance.

Quantitative PCR is a widely used technique for analyzing the expressions of specific drought-resistant genes. Researchers can determine how the expression of genes like *ZmDREB2/2.5/A* [80,81,82], *ZmAREB*, and *ZmP5CS* [83] change in response to drought stress by analyzing the mRNA levels of these genes. A more thorough method that enables the simultaneous profiling of thousands of genes is RNA sequencing, or RNA-Seq. In maize under drought stress, RNA-Seq has been utilized to find genes that are differently expressed and to shed light on changes in gene expression around the world. Research has demonstrated that during drought stress, genes related to ABA signaling, osmotic control, and ROS scavenging are significantly increased [84]. The expression of genes in maize that respond to drought has been examined using microarrays. According to these findings, a complex regulatory network that includes stress proteins, transporters, and transcription factors is induced during drought and aids maize plants in adjusting to water shortages.

Utilizing the natural genetic variation of plant quantitative traits linked to stress tolerance—where quantitative genetics plays a crucial role through classical and molecular breeding—is the primary genetic strategy for improving multiple-stress tolerance. Genetic areas linked to drought tolerance have been found using QTL analysis. Certain loci have been connected to important characteristics, such as leaf water potential, stomatal conductance, and root depth. Through the overexpression of genes resistant to drought, transgenic techniques have been used to improve drought tolerance. For instance, it has been demonstrated that increasing *ZmP5CS* and other genes linked to drought enhances tolerance to drought in maize plants [84].

**Table 1:** List of the genes and their role identified by researchers in recent years.

S. No	Gene	Details	Function/s	Reference
1	*ZmHB53*	Homeodomain-leucine zipper I (HD-Zip I) transcription factors (TFs)	ABA receptor *ZmPYL4*	[85]
2	*ZmPHR1*	Transcription factor	phosphorus homeostasis	[86]
3	*ZmTIP2;3*	Tonoplast intrinsic protein	arbuscular mycorrhiza fungi symbiosis	[87]
4	*ZmSCE1a*	E3 SUMO ligase	enhancing the stability of *ZmGCN5*	[88]
5	*ZmNAC55*	Trnacription factor	negatively regulate drought stress via increasing *ZmHOP3* expression in maize	[89]
6	*ZmMIK2-ZmC2DP1*	Kinase 2 proteins	negative regulatory module in maize drought- and salt-stress responses	[90]
7	*ZmCML3*	Calmodulin-like proteins (CMLs)	through increasing proline (Pro) content	[78]
8	*ZmGA20ox3*	Loss-of-function mutations of GA biosynthesis enzyme	significantly increased ABA, JA, and DIMBOA levels in mutants	[91]
9	*ZmEULD1b*	*Euonymus europaeus* (EUL) related lectin family,	stomatal development and promotes water-use efficiency	[92]
10	*ZmMYB39*	Transcription factor	stomatal development and promotes water-use efficiency	[93]
11	*ZmGLYI-8*	Glyoxalase I (GLYI)	Overexpressed in model plants	[94]
12	*ZmbHLH47-ZmSnRK2.9*	Transcription factor	ABA response and drought tolerance	[95]
13	*ZmAPX2*	Ascorbate peroxidase 2	reducing ROS content	[96]
14	*ZmSK1*	Glycogen synthase kinase 3 (GSK3)-like kinases	reduces drought tolerance in maize	[97]
15	*ZmDST44*	Drought and salinity tolerance (DST) gene	positive regulator of drought tolerance (*ZmmiR139* regulates *ZmDST44* by cleaving its mRNA)	[98]
16	*ZmPL1*	Phylloplanin-like	negatively regulates drought tolerance in maize (CRISPER-cas9)	[81]
17	*ZmC2H2-149*	Cys(2)/His(2) zinc-finger-proteins (C2H2-ZFPs)	repressing *ZmHSD1* in maize (negative regulator)	[99]
18	*ZmPRX1*	Peroxidase genes	promoting root development and lignification	[100]
19	*ZmSUS1*	Sucrose synthase (SUS)	regulating sucrose metabolism and increasing soluble sugar content	[101]
20	*ZmGRAS15*	GRAS transcription factor	regulating primary root length at the seedling stage	[102]
21	*ZmCYB5-1*	Cytochrome b5 proteins (CYB5s)	negative regulator of drought stress	[103]
22	*ZmHsf28*	Transcription factors	*ZmSnRK2.2-ZmHsf28-ZmJAZ14/17* module is identified to regulate drought tolerance through coordinating ABA and JA signaling	[104]
23	*miR166e/ZmATHB14*	Micro RNAs	*miR166e-ZmATHB14* module regulates drought tolerance	[105]
24	*ZmSNAC06*	NAC transcription factor family	hypersensitivity to abscisic acid (ABA)-positive regulator	[106]

## 5. Heat Resistance in Maize: Effects and Mechanisms

### 5.1. Impact of High Temperatures on Maize Physiology

**Figure 4:**
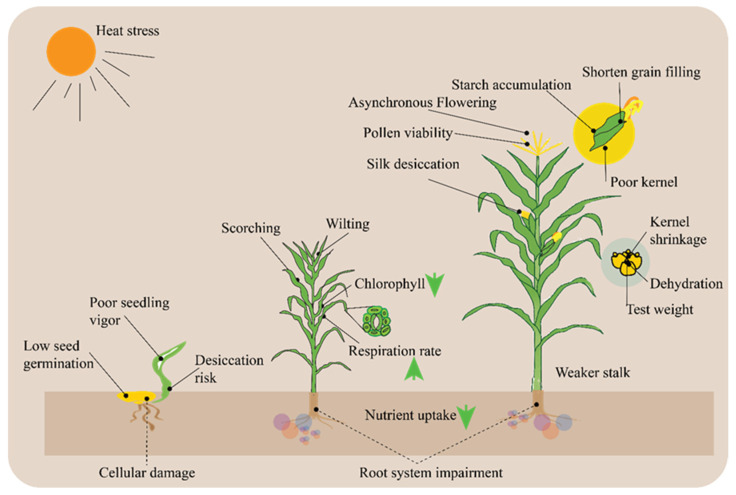
Comprehensive overview of heat stress on maize plants. Three different plants are depicted to show different stages of growth and development. Heat stress can lead to issues such as poor seedling vigor, desiccation risk, and cellular damage during early development. As the plant matures, it may experience chlorophyll degradation, increased respiration rates, nutrient uptake impairment, and root system damage. Later stages show effects like asynchronous flowering, pollen viability issues, silk desiccation, and ultimately poor kernel formation, kernel shrinkage, and weaker stalks, which result in shortened grain filling and reduced test weight.

High temperatures can profoundly affect maize physiology, particularly during critical growth stages such as blooming and grain filling. Maize, as a warm-season crop, thrives across a wide range of temperatures. However, when it is subjected to temperatures higher than its ideal range, which is typically between 30 °C and 35 °C, physiological processes are interfered with, which results in decreased growth and productivity. Developing methods to reduce heat stress and increase crop resilience in the face of climate change requires an understanding of how high temperatures affect maize physiology. In maize, high temperatures, particularly those above 35 °C, can inhibit seed germination and seedling establishment. Heat stress on maize plants at different stages of growth can affect growth and development (Figure 4). From seed germination to grain filling, heat stress can lead to a range of adverse effects, including poor seedling vigor, desiccation risk, wilting, chlorophyll damage, asynchronous flowering, pollen viability issues, silk desiccation, shortened grain filling, poor kernel development, kernel shrinkage, dehydration, weaker stalks, and impaired nutrient uptake (Figure 4). These factors collectively result in reduced crop yield and quality, highlighting the critical need for strategies to mitigate heat stress in agricultural practices.

Defective seed imbibition, often caused by high temperatures during germination, can hinder the activation of metabolic processes essential for seedling emergence. Additionally, high temperatures can interfere with the enzyme activity necessary for the decomposition of seed reserves, which hinders the seed’s capacity to sprout [107]. Both root and shoot growth may be impeded by rising temperatures. As plant’s ability to absorb water and nutrients may be limited if the root system does not grow sufficiently to support it. High temperatures can also harm the cell membranes in developing tissues, which inhibits cell elongation and division two processes essential to early plant growth [108]. Maize physiology is significantly affected by high temperatures, especially with regard to photosynthesis, reproductive development, and water relations. Reduced growth and yield are the results of oxidative stress, membrane damage, and water deprivation, particularly during crucial reproductive periods. Developing methods to reduce heat stress, such as breeding heat-tolerant cultivars and putting agronomic techniques like irrigation control and shade cover into place, requires an understanding of these physiological reactions. Enhancing maize’s ability to withstand heat will be crucial to maintaining global food security as climate change continues to increase the frequency and severity of heat events.

### 5.2. Heat Tolerance Mechanism

The physiology of maize is negatively impacted by heat stress, particularly at crucial developmental stages like flowering and grain filling, which lowers yield. In order to survive heat stress, maize plants have developed several coping strategies that enable them to preserve cellular integrity, continue growing, and maximize reproductive success in adverse circumstances. The production of heat shock proteins (HSPs) and the preservation of cellular homeostasis are two important processes for heat stress resistance. These systems play a crucial role in protecting the plant from oxidative stress, protein denaturation, and other damage caused by high temperatures. The function of heat shock proteins (HSPs) in protein folding is related to their role in heat tolerance. The class of molecular chaperones known as heat shock proteins (HSPs) is essential for shielding cells from harm while they are under heat stress. These proteins support healthy protein folding under stressful circumstances, aid in the refolding of damaged proteins, and guard against the denaturation of cellular proteins. Heat shock factors (HSFs), which function as transcription factors to start the synthesis of HSPs, are activated in response to high temperatures, increasing the expression of HSPs. Certain HSPs are upregulated in maize in response to heat stress. For example, it has been demonstrated that HSP70 plays a crucial role in heat tolerance, helping to preserve protein homeostasis and shield cellular structures from heat [108]. Furthermore, another heat-induced chaperone, HSP101, contributes to heat tolerance by aiding in the recovery of plants from heat-induced damage, which improves their ability to survive and develop in hot conditions [109]. The stability of proteins, the integrity of cellular structures, and the general equilibrium of ions and metabolites are all at risk under heat stress. Maize plants use a variety of tactics, mediated by HSPs, antioxidants, and modifications in cellular metabolism, to preserve cellular homeostasis and survival. The lipid structures in membranes can be disrupted by high temperatures, which can impair the activity of proteins and enzymes that are membrane-bound and cause membrane fluidity. Under heat stress, maize plants modify their lipid composition by producing unsaturated fatty acids for preserving the integrity of their membranes. This modification stops cellular contents by keeping the membrane steady [110]. Recent studies for the genes and transcription factors identified were listed in Table 2.

## 6. Emerging Technologies for Enhancing Drought and Heat Resistance in Maize: From a CRISPR Perspective

**Figure 5:**
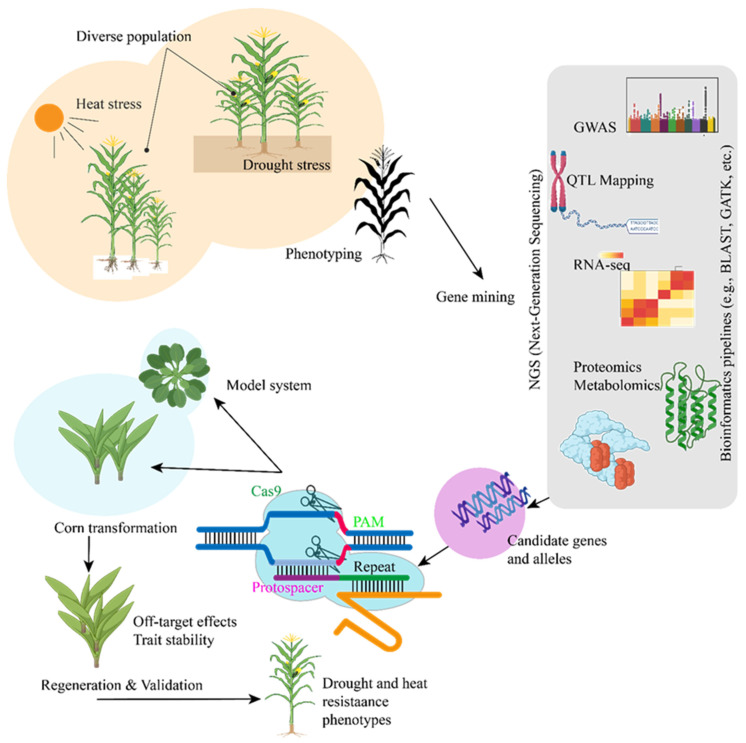
Model to illustrate the application of CRISPR-Cas9 technology in maize improvement to enhance resistance against heat and drought. The process involves targeting specific genes using guide RNA (gRNA) designed to bind to the protospacer region, enabling precise genome editing for stress tolerance enhancement.

Several genetic engineering methods have been used to create maize cultivars that can withstand drought and heat. To maximize drought resistance, these strategies can be roughly divided into three categories: gene editing, introducing target genes, and manipulating multiple genes. The overexpression of genes resistant to drought is one of the most popular strategies for enhancing drought tolerance. Researchers can improve maize’s resistance to water stress by introducing genes like *ZmDREB2*, *ZmP5CS*, and *ZmSOD* into the plant’s genome. For example, it has been demonstrated that overexpressing *ZmDREB2* increases maize production during drought by triggering downstream stress-response genes. Alternatively, genes that prevent drought tolerance can be silenced via RNA interference (RNAi). For instance, prolonging the growth period of maize under drought stress can increase productivity by silencing genes linked to stress-induced premature senescence. The CRISPR-Cas9 genome-editing technique provides a more accurate method for changing genes linked to drought tolerance [117]. This method enables the targeted modification of particular genes in maize, such as *ZmABF* or *ZmDREB2* regulation, to improve the plant’s resistance to drought. CRISPR can be used to eliminate genes that negatively impact water use efficiency, such as those associated with excessive transpiration or premature senescence. For example, CRISPR-Cas9-mediated editing of *ZmPL1* gene showed negative role of Phylloplanin-like 1 in drought tolerance [118]. There are QTLs linked to heat tolerance in maize, and certain loci have been linked to features like pollen viability, photosynthetic efficiency, and thermotolerance. Increased heat resistance is linked to QTLs such as qHT1 on chromosome 2. Genetic engineering aims to introduce heat-resistant genes into maize for improved resilience. For instance, the capacity to tolerate high temperatures, particularly during reproductive development, can be improved by overexpressing *HSP70* and other genes linked to heat stress [119]. Even expression analysis of the *Hsf* and *Hsp70* has showed positive regulation of stress in maize [120]. Recent advancements in omics technologies (Figure 5) have provided researchers with a comprehensive understanding of how maize responds to environmental stressors. While proteomic and metabolomic investigations shed light on changes in proteins and metabolites that occur during stress, transcriptomic analyses assist in identifying genes that are differentially expressed under drought and heat stress conditions. By focusing on important genes involved in stress responses, CRISPR-Cas9 genome-editing technology has the potential to produce maize variants with increased resistance to a variety of pressures (Figure 5) [117,121].

Stress-resilient maize varieties may be developed more quickly thanks accurate and effective methods [122]. Because of its accuracy and effectiveness, CRISPR-Cas9 is a potent tool for creating maize cultivars with specific drought-resistant characteristics without adding extraneous genes that can raise regulatory issues. In certain situations, a single gene might not offer enough resistance to drought in the field. To get around this, several genes implicated in several drought tolerance systems, including osmotic control, stress signaling, and antioxidant defense, are introduced using multigene engineering. This method can simultaneously improve multiple physiological processes, leading to more resilient drought-resistant cultivars. For example, maize plants that have been modified with both *ZmDREB2* [80,123] and *ZmP5CS* show increased drought tolerance and better osmotic adjustment.

Emerging biotechnology advancements are essential in improving maize’s resilience to environmental challenges like heat, drought, and salinity as a result of climate change. Of them, the gene-editing technique known as CRISPR-Cas9 is one of the most promising means of producing crops that are more resilient to these difficulties. A groundbreaking technology in genetic engineering, CRISPR-Cas9 enables precise DNA changes at specified sites in an organism [124,125]. Because it allows for the targeted alteration of genes involved in stress-response pathways without introducing foreign DNA, this is very useful for enhancing crop resilience (Figure 5). CRISPR has several advantages over conventional genetic alteration methods, including accuracy, speed, and affordability. Through the targeting of genes that control important stress-response pathways, CRISPR-Cas9 can be used to increase maize’s resistance to abiotic stress like heat, drought, and salinity. Researchers can make maize more resilient to harsh environments by suppressing or deleting genes that are sensitive to stress. CRISPR-Cas9 is one of the emerging technologies that has the potential to improve maize’s resistance to environmental challenges like heat, drought, and salinity. New possibilities for developing more adapted crops to a changing environment are made possible by the capacity to precisely modify genes linked to stress-response pathways [2,126]. While CRISPR-Cas9 has revolutionized crop improvement by enabling precise genetic modifications, it is not without limitations [127]. One major concern is the potential for off-target effects, where unintended genomic changes may occur, leading to unpredictable phenotypes or undesirable traits [128]. Additionally, regulatory hurdles pose a significant challenge, as many countries classify gene-edited crops under strict GMO regulations, slowing down their adoption and commercialization. To address these issues, emerging genome-editing techniques such as prime editing and base editing offer more precision and control, minimizing off-target activity and enabling subtle, targeted modifications without inducing double-strand breaks, thereby enhancing both safety and regulatory acceptability.

## 7. Conclusions

Breeding programs today face the dual challenge of increasing crop yields to meet global food demands while enhancing resilience to climate-induced stresses. In crops like maize, achieving this balance is critical due to the increasing frequency of extreme weather events such as drought, heat, and flooding. Advances in biotechnology, including gene editing (CRISPR-Cas9), marker-assisted selection, and genomic tools, offer powerful strategies to accelerate the development of high-yielding, stress-tolerant varieties. However, for these innovations to have a lasting impact, their scalability across diverse agroecosystems must be carefully considered. CRISPR-edited maize lines developed under controlled conditions must undergo rigorous testing in varied environments to ensure stable performance across different climates, soils, and management practices. Local adaptation remains key; therefore, integrating edited traits into region-specific germplasm is essential for successful deployment. Furthermore, multi-trait breeding approaches and strong collaborations between researchers, breeders, and policymakers will be vital to translate laboratory breakthroughs into field-level solutions. Utilizing genetic diversity from landraces and wild relatives can further enhance the adaptability of elite lines. Ultimately, combining conventional breeding with modern biotechnological tools will enable the development of maize varieties that not only thrive under optimal conditions but also withstand the growing uncertainties posed by climate change—ensuring food security for future generations.

## Figures and Tables

**Table 2:** List of genes identified in recent years with basic studies related to drought resistance in maize.

Gene	Details	Mechanisms/Method	References
*ZmHsp18*	ZmHsp20 gene family	Gene family-based perfection	[111]
*ZmENO1*	Enolase (ENO, 2-phospho-D-glycerate hydrolyase)	Antioxidant enzyme activities and osmotic regulation	[112]
*ZmDnaJ genes*	HSP40s	Correlation between heat stress tolerance and the regulation of genes	[113]
*ZmDnaJ-ZmNCED6*	Heat shock protein	Involved in ABA signal transduction pathways	[114]
*cpSRP43*	CMT2 and cpSRP43	CHROMO domain family genes	[115]
*ZmDnaJ96*	DnaJ/HSP40 gene family	Increased antioxidant enzyme activity	[116]
*ZmATL10 and AtATL27*	ATL family		[49]

## Data Availability

Not applicable.
